# Assessment of DNA Damage and Telomerase Activity in Exfoliated Urinary Cells as Sensitive and Noninvasive Biomarkers for Early Diagnosis of Bladder Cancer in Ex-Workers of a Rubber Tyres Industry

**DOI:** 10.1155/2014/370907

**Published:** 2014-04-30

**Authors:** Delia Cavallo, Valentina Casadio, Sara Bravaccini, Sergio Iavicoli, Enrico Pira, Canzio Romano, Anna Maria Fresegna, Raffaele Maiello, Aureliano Ciervo, Giuliana Buresti, Wainer Zoli, Daniele Calistri

**Affiliations:** ^1^Department of Occupational Medicine, INAIL-Italian Workers' Compensation Authority, Research Area, Monteporzio Catone, 00040 Rome, Italy; ^2^Bioscience Laboratory, Istituto Scientifico Romagnolo per lo Studio e la Cura dei Tumori, Meldola, 47014 Forlì-Cesena, Italy; ^3^Department of Traumatology, Orthopaedics and Occupational Medicine, University of Turin, 10126 Turin, Italy

## Abstract

The aim of the present study was to identify sensitive and noninvasive biomarkers of early carcinogenic effect at target organ to use in biomonitoring studies of workers at risk for previous occupational exposure to potential carcinogens. Standard urine cytology (Papanicolaou staining test), comet assay, and quantitative telomerase repeat amplification protocol (TRAP) assay were performed in 159 ex-rubber workers employed in tyres production and 97 unexposed subjects. In TRAP positive cases, a second level analysis using FISH (Urovysion) was done. Cystoscopy results were available for 11 individuals whose 6 FISH/TRAP/comet positive showed in 3 cases a dysplastic condition confirmed by biopsy, 1 comet positive resulted in infiltrating UBC to the biopsy and with hyperplasia and slight dysplasia to the urinary cytology, 1 comet positive resulted in papillary superficial UBC to the biopsy, 1 FISH/TRAP positive showed a normal condition, and 2 TRAP positive showed in one case a phlogosis condition. The results evidenced good concordance of TRAP, comet, and FISH assays as early biomarkers of procarcinogenic effect confirmed by the dysplastic condition and UBC found by cystoscopy-biopsy analysis. The analysis of these markers in urine cells could be potentially more accurate than conventional cytology in monitoring workers exposed to mixture of bladder potential carcinogens.

## 1. Introduction

Bladder cancer is a frequent and very aggressive malignant tumor representing during 2011 the fourth most common malignancy in men and the ninth in women [1]. More than 90% of bladder malignancies are urothelial bladder carcinomas (UBC) characterized by proliferation of inner superficial layer of urinary bladder (urothelium) that is constantly exposed to metabolites and other chemicals through contact with urine [2, 3]. Smoking is the most common risk factor for UBCs and occupational exposure to aromatic amines and polycyclic aromatic hydrocarbons are other important risk factors [4]. Of particular aetiological importance is a history of exposure to chemical substances, which, as carcinogens or cocarcinogens, may lead to the development of carcinoma with a latency of up to 30 years [2].

Workers employed in rubber industry appear to have a significant excess cancer risk of the urinary bladder associated with occupational exposure to complex mixture of chemicals [5–9]. The International Agency for Research on Cancer (IARC) classified “rubber manufacturing” as carcinogenic for humans (group 1) on the basis of epidemiological reports indicating excess of cancer risk at several sites (bladder, lung, stomach, and blood) [10, 11]. The bladder cancer risk in rubber workers has been ascribed principally to 2-naphthylamine present as contaminant of the phenyl-2-naphthylamine, used in the past as antioxidant, but also to numerous other chemicals including other antioxidant aromatic amines, acrylonitrile, 1,3-butadiene, styrene, polycyclic aromatic hydrocarbons (PAHs) used as reinforcing agents, organic accelerators, activators, vulcanization agents, and substances produced during high temperature processes (mixing and vulcanization) of rubber production many of which are known to be genotoxic [12].

Early detection of bladder cancer in this job category at risk for previous exposure to bladder potential carcinogens is particularly important for improved prognosis and long-term survival.

Detection and monitoring of urinary bladder cancer are usually done by urine cytology, cystoscopy, and histology [13, 14]. However these methods are highly subjective, expensive, and invasive and often are not able to reveal low-grade UBC at the first stage of transformation (urine cytology) or flat bladder cancer in situ and bladder cancer which remain below the mucosa surface (cystoscopy) [15].

Noninvasive methods which are able to compete with cystoscopy and to implement cytology diagnostic accuracy are still needed.

In recent years, some noninvasive tests performed on voided urine have been developed, approved by FDA [16], and are commercially available: nuclear matrix protein 22 (NMP22) levels, Fluorescent In Situ Hybridization (FISH) UroVysion assay and immunocyt [2, 14, 16, 17]. Other urinary biomarkers such telomerase activity (TA) levels by Telomeric repeat amplification protocol (TRAP) assay [18, 19], DNA damage in exfoliate urinary cells by comet assay [20], and DNA methylation markers are being investigated [16] and seem to be good candidates for early detection of bladder cancer. However these biomarkers require being validated in further studies on subjects at risk.

The use of noninvasive and highly sensitive test in combination with standard assays can improve early diagnosis of new early stages bladder cancer and could be particularly useful to screen professionally high-risk groups like rubber workers.

It has been demonstrated that comet assay is able to sensitively reveal early, still repairable, DNA damage and furnish useful information on early effects induced by exposure to genotoxic substances [21–23].

Many recent studies have also showed that telomerase activity quantification by TRAP assay in urine sediments, alone or in combination with a second level analysis, has an important role in distinguishing cancer patients from healthy individuals and also symptomatic patients [15, 18].

In our study, we investigated in exfoliated urothelial cells of ex rubber workers employed in tyre production, the presence of DNA damage by comet assay, and the telomerase activity levels by TRAP assay, combining them with FISH analysis. The results are compared with the commonly used cytology and cystoscopy used as gold standard.

The aim is to identify sensitive and noninvasive biomarkers of early carcinogenic effect at target organ to use in biomonitoring studies of workers at risk for previous occupational exposure to potential carcinogens.

## 2. Materials and Methods

### 2.1. Subjects

The study was carried out on 159 ex-rubber workers aged 31 to 81 years, with mean age (±SD) 60 ± 12 years, employed in tyres production, at variable times in the period between 1962 and 2006, potentially exposed in the period 1962–1980 to the potential carcinogen phenyl-2-naphthylamine, in the period 1981–1995 to aromatic amines (IPPD, 6-PPD), and in entire period to other chemicals such as carbon black, 1,3-butadiene, styrene, polycyclic aromatic hydrocarbons (PAHs) used as reinforcing agents, organic accelerators, activators, and vulcanization agents. A group of 97 unexposed healthy subjects aged 30 to 83 years (mean age 57 ± 12 years) were used as controls. The Local Ethics Committee approved the study protocol and all subjects gave written informed consent to take part in the study. Anagraphic, clinical, working information and lifestyle habits (smoking, dietary habit, and alcohol consumption) were obtained from a questionnaire administered by specialized medical personnel.

**Figure 1:**
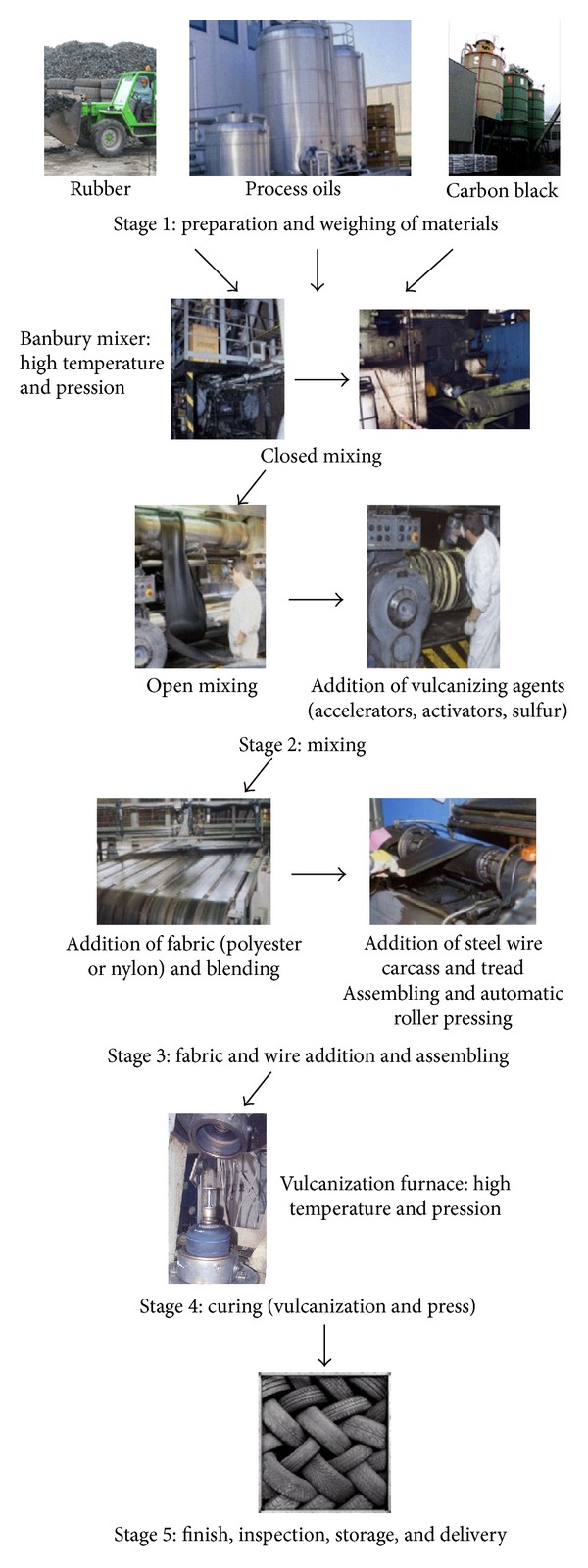
Working stages of tyre production process. The process starts with weighing rubber, process oils, carbon black, and other additives. In the mixing stage, the raw materials are mixed together and heated. Then assembling with reinforcements occurs and finally the tyre undergoes vulcanization in curing presses. The higher risk in developing bladder cancer in stages 1, 2, and 4 is due to the presence of potentially carcinogenic compounds. The last stage comprises inspection, testing, and delivery.

On the basis of the performed activities, the working cycle has been divided into 4 stages (Figure 1): stage 1 (preparation and weighing of raw materials), stage 2 (mixing raw materials in Bambury, cutting the compound/immersion cooling tanks and rubberized chassis production), stage 3 (pneumatic assembly and installation), stage 4 (vulcanization), and phase 5 (checking, storage, and transportation of the finished product). The steps 1, 2, and 4 of the production cycle are at greater risk of exposure to the presence of carbon black, antioxidants such as aromatic amines, IPA, additives, curatives, accelerants, and activators.

Since quantitative measurements of past exposure to amines and other chemicals were not available, we reconstructed the past exposure of ex-workers using matrix job/exposure. In particular for each worker, we considered the following working information: department, task and seniority in specific work stage, year of the beginning, and end of exposure. On the basis of these information, we identified five classes of risk for previous exposure to amines and other potentially carcinogenic chemicals (considering the period 1962–1980 at greater risk for the presence of aromatic amines such as phenyl-2-naphthylamine and other chemicals no longer allowed and for the probable lack of personal or environmental protective measures) (Table 1).

### 2.2. Urine Collection

The urine samples from both workers and control subjects were collected freshly in the morning of the first two days of week and divided in three aliquots. One aliquot was immediately processed for cytological analysis, while the other were treated to be sent to the laboratories where comet assay and TRAP analysis were performed.

In particular for TRAP analysis, 3 mL of urine samples was centrifuged (5000 rpm for 10 min at 4°C) within 1 to 3 hours of urine sample collection, washed once in PBS buffer, resedimented by centrifugation (5000 rpm for 10 min at 4°C), frozen at −80°C, and sent to the Laboratory where the analysis was performed. For comet assay, one 50 mL aliquot of each freshly voided urine sample was centrifuged (1500 rpm for 10 min), suspended in 20 mL of PBS buffer, and transferred within 24 hours, at 4°C and in the dark, to the laboratory where the analysis was performed. In the cases analysed also by FISH, in accordance with the manufacturer's instruction (UroVysion; Abbott/Vysis, Downers Grove, IL), one aliquot of at least 33 mL of urine was used and sent to the laboratory where the FISH was performed.

### 2.3. Comet Assay

Comet assay with appropriate modifications, due to the specific cell type used, was performed to evaluate early DNA damage in exfoliated urinary cells. Briefly, immediately before performing comet assay, exfoliated urinary cells were washed in the PBS buffer solution and then suspended in about 100 *μ*L of the same buffer.

Ninety microliters of normal-melting agarose (NMA) 1% in PBS at 50°C was layered onto bond gel film (Sigma), immediately covered with a coverslip, and allowed to solidify at 4°C for 5 min. The coverslip was then removed and about 80 *μ*L of exfoliated cells suspension was mixed with 80 *μ*L of low-melting agarose (LMA) 0.7% in PBS at 37°C and layered on top of the film. A coverslip was added and the film was left to solidify at 4°C for 10 min. The coverslips were taken off and the films were layered onto glasses slides and treated with 70 *μ*L/slide of Proteinase K (1 mg/mL) for 2 h at room temperature, in order to digest proteins and inactivate nucleases responsible for degrading DNA. Then the slides were bathed in freshly prepared lysis solution (2.5 M NaCl, 100 mM Na_2_EDTA, 10 mM Tris with 1% Triton X-100 and 10% DMSO added fresh) overnight, in the dark at 4°C. The slides were removed from the lysing solution and placed in a horizontal gel electrophoresis tank filled with fresh alkaline buffer (1 mM Na_2_EDTA and 300 mM NaOH, pH 13) for 40 min at 4°C to allow denaturing and unwinding the DNA and the expression of alkaline-labile sites.

Electrophoresis was done in the same buffer at 25 V and 300 mA for 20 min to allow the damaged DNA or fragments to migrate towards the anode. The slides were then washed three times with Tris HCl 0.4 M for 5 min and stained with 50 *μ*L ethidium bromide (10 *μ*g/mL). Slides were examined by eye at 200x magnification under a fluorescence microscope. An undamaged cell appeared as a nucleoid and a cell with damaged DNA as a comet. About 1000 cells from each case were examined for presence of comets by an experienced observer and the percentage of comets on total cells was calculated.

To evaluate the extent of DNA damage, images of 50 randomly selected comets were acquired and analyzed from each sample with specific image analyzer software (Delta Sistemi, Italy) that allows measuring specific parameters at level of single comet image.

Measurements of comet parameters were % DNA in the tail, tail length, and tail moment (the product of relative tail intensity and length, used as a measure of DNA damage). The mean tail moment (Tm) and % DNA in the tail of 50 comets had been calculated for each subject.

The mean values Tm = 30 and tail DNA% = 26 were selected as cut off normal values. For each subject the presence of mean values of Tm and tail DNA% ≥ than cut off normal values were considered indicative of DNA damage.

### 2.4. TRAP Assay

Pelleted cells were resuspended in 200 *μ*L of ice cold TRAP lysis reagent [24] and left on ice for 30 min.

Cell lysates were centrifuged at 10000 ×g for 20 min at 4°C and the supernatant was stored at −20°C. Urine sample aliquots containing 1 *μ*g of protein lysate were used for the TRAP assay. As previously described [25], telomerase products were evaluated on fluorescent electropherograms and the area underlying the different peaks was calculated. To obtain semiquantitative levels of telomerase activity, an internal telomerase assay standard (ITAS; 25 attograms), amplified by the same 2 primers used for the telomerase activity assay, was included in the TRAP buffer. For the correct evaluation of telomerase activity values of each sample, a standard curve was used. In particular, protein concentrations corresponding to 10, 30, 100, 300, 1000, and 3000 cells of a human bladder cancer line (MCR) were analyzed in each assay and used as the reference curve. To obtain quantitative evaluations of telomerase activity, the areas of each sample were also normalized to the 150-base pair ITAS peak. The relative telomerase activity per cell for each sample is presented as the percentage of the ratio of TRAP ladder/ITAS per cell versus the value of MCR and expressed in arbitrary enzymatic units (AEUs). All experiments were performed in duplicate, and when variations were greater than 15%, observed in about 10% of cases, a third analysis was performed. Telomerase activity was expressed as a continuous variable in all analyses.

### 2.5. FISH Analysis

Exfoliated urothelial cells were collected from each urine aliquot by cytospin centrifugation (1200 ×g for 5 min). Cells were fixed in Carnoy's fixative for 20 min and dehydrated for 2 min each in ethanol scalar dilutions (70%, 85%, and 100%) after which the slides were heated at 45°C for 2-3 min and stored at 4°C for a maximum of 1 month. Hybridization was carried out according to the manufacturer's instructions (UroVysion; Abbott/Vysis). The probe mixture consisted of fluorescently labeled probes targeted on the pericentromeric regions of chromosomes 3 (CEP3 Spectrum Red), 7 (CEP7 Spectrum Green), and 17 (CEP17 spectrum Aqua) and on band 9p21 locus (LSI 9p21 Spectrum Gold) over each target area; 3 *μ*L of the probe solution was applied. Target DNA and FISH probe were codenatured at 73°C for 2 min using a commercial hybridization system (HYBrite; Abbott/Vysis) and subsequently incubated at 39°C overnight. 4′,6-Diamidino-2-phenylindole was used for counterstaining and cells were viewed using a fluorescent microscope (Axioscope, Zeiss, Göttingen, Germany). All slides were scanned and the number of CEP3, CEP7, CEP17, and 9p21 signals in the cells was determined. The presence of at least 25 cells per slide was required to consider a sample as evaluable, and two signals for each chromosome were required to consider a cell as normal. The presence of four or more cells with gains for two or more chromosomes (3, 7, and 17) in the same cell or 12 cells or more with zero 9p21 signals was the criterion to consider a specimen as positive. Slides were evaluated independently by two experts blinded to the patient's history and histological findings.

### 2.6. Cytology

Standard urine cytology (Papanicolaou staining test) for the assessment of markers indicative of bladder cancer was performed. Briefly, urinary cells were prefixed in ethanol, harvested by cytocentrifugation, spread on slide, dried, stained with Papanicolaou staining, and then analyzed by an experienced observer, in order to identify cells morphology and presence of infective agents.

### 2.7. Statistical Analysis

Statistical analysis was performed with SPSS 16.0 package (SPSS Inc., IL, USA). We analysed preliminarily the presence of possible significant differences between exposed subjects and controls for confounding factors: by nonparametric Mann-Whitney *U*-test for age and by chi square test for smoking habits, alcohol intake, drug assumptions, diagnostic radiation exposure, and dietary habit. Nonparametric Mann-Whitney *U*-test was used to test the significance of differences in the studied biological parameters (tail moment and TRAP mean values) between exposed and unexposed subjects, Kruskal-Wallis with post hoc Bonferroni test was used to test the significance of differences in the studied biological parameters among the different classes of risk, and *P* ≤ 0.05 was considered significant. Chi square test followed by exact Fisher test when expected frequencies were <5 was used to analyze the significance of association between positivity frequency at comet assay or TRAP and exposure also in relation to the class of risk. The contingency table was used to analyze the differences of positive/negative comet test and TRAP results both in exposed subjects and controls and the statistical significance has been assessed with chi square test.

Pearson's correlation coefficients (*r*) were calculated to evaluate the relationship between TRAP and comet results both in exposed subjects and controls.

## 3. Results

No statistically significant differences were evidenced for confounding factors such as smoking habits, diagnostic radiation exposure, and drug assumptions between rubber workers and controls while significant differences were found for age and dietary habit (grilled and smoked food intake, alcohol intake) (Table 2).

For comet assay, tail moment (Tm) indicative of DNA damage was evaluated and subjects with mean values ≥ than cutoff normal value (Tm = 30) were considered positive. The mean Tm values were slightly higher in rubber workers than in controls (16.81 versus 14.11) and DNA damage was present in 10% (16/159) workers in respect to 2% (2/97) of controls. Tm values were significantly higher in the subjects included in the highest class of risk compared with the other classes of risk and with the controls (*P* = 0.023). The rubber workers with DNA damage were included mainly (13/16) in the class at the highest risk showing a significant association with exposure (exact Fisher test *P* value = 0.020 in the highest class of risk and *χ* ^2^ *P* value = 0.015 in exposed group versus controls) (Table 3).

TRAP analysis showed results ranging from 5 to 123 arbitrary enzymatic units (AEU), with median values of 37 AEU in ex-exposed and 34 AEU in controls. Considering positive TRAP values ≥50 AEU, the subjects with TRAP positive levels were 26/159 (16%) in exposed group and 15/97 (15%) in controls. The rubber workers with TRAP positive levels were included mainly (18/26) in the class at the highest risk (Table 3). Concordant comet and TRAP results were observed in 139/159 (87%) ex-exposed group with positivity in 11 workers belonging prevalently (10/11) to the classes (4 and 5) with at least 10 years in mixing and curing activities, at higher risk for previous exposure to carcinogens, showing significant concordance between positive/negative comet and TRAP results with a chi square *P* value <0.001. In addition, correlation analysis between comet and TRAP results showed statistical significance between Tm and AEU values only in workers group (Pearson's correlation index *r* = 0.225, *P* value =0.005).

FISH analysis was performed on 19 TRAP positive cases and showed positive concordant results in 14/19 (74%) cases (Table 3), 9 of which were positive also for comet assay.

Cytological analysis showed hyperdysplastic features only in one rubber worker, included in the class at highest risk, resulted positive also at comet test with confirmed diagnosis of infiltrating UBC by biopsy (Table 4), while negative results for atypical cells were observed in the remaining cases.

Among subjects with FISH, TRAP, or comet positive results, 11 subjects agree to perform cystoscopy. The cystoscopy-biopsy identified in 3 subjects (with FISH, TRAP and Comet positive results) a dysplastic condition; in 1 comet positive subject infiltrating UBC (resulted with hyperdisplasia/slight dysplasia to the urinary cytology) and in 1 comet positive subject a papillary superficial UBC. The remaining subjects (3 FISH/TRAP/comet positive and 2 TRAP positive) showed a normal or phlogosis condition (Table 4).

## 4. Discussion

This study aims at identifying a set of sensitive and noninvasive techniques for early diagnosis of bladder lesions. Here a genotoxicity test, such as comet assay, and the quantitative TRAP assay are combined with FISH analysis. The results are compared with the commonly used cytology and cystoscopy used as gold standard.

Cytology test is very specific (a positive result is highly indicative of bladder cancer) but suffers from low sensitivity (a negative result does not exclude the diagnosis of cancer, particularly for cancer at first stages); comet test is able to identify early DNA damage that could increase the risk of cancer and TRAP analysis seems to be able to sensitively detect telomerase activity levels in superficial urothelial cell carcinoma [15, 18, 26].

Comet assay on the ex-rubber workers employed in tyre production, exposed in the past (1962 to the early 90) to aromatic amines (phenyl-2-naphthylamine, IPPD, 6-PPD, DBPPD), showed that tail moment values are significantly higher for the subjects included in the highest class of risk compared with the other classes of risk and controls. Moreover we found the presence of DNA damage (established on the basis of cutoff normal values for TM) in the 10% of exposed workers (16/159 subjects) in respect to the 2% of control group (2/97 subjects).

We found DNA damage in workers employed for 25–30 years in work stages 1-2-4, corresponding to the production functions “preparation of materials,” “mixing,” and “vulcanization,” areas with potential hazard for exposure to a wide range of chemical compounds used in rubber manufacturing. The presence of higher DNA adduct levels in exfoliated bladder cells of rubber workers involved in the production functions “mixing” and “curing” was demonstrated by Vermeulen et al. [27] suggesting for these work stages potential exposure to compounds that can interact with DNA in urothelial cells. Our comet results seem to support the above study showing a relationship between past exposure in specific work stages of rubber production and genotoxic effects. On 8 ex-exposed with positive comet assay (6 of which resulted in being positive also at TRAP and FISH analyses), the cystoscopic examination was performed showing in 3 cases a dysplastic condition, in 2 cases the presence of urothelial cancer, and in 3 cases a normal condition. Therefore, 5/8 individuals, submitted to cystoscopy, showed conditions predisposing to cell transformations.

These findings demonstrated the suitability of urinary cells, representing the target tissue of exposure to potential bladder carcinogens used in rubber industry, and the higher sensitivity of comet assay in respect to standard cytology in identifying early genotoxic effect in agreement with the study of Fracasso et al. 2004 [20] performed on patients with clinical suspicion of bladder cancer or urological disorders. Comet assay on exfoliated urinary cells could represent a sensitive and noninvasive biomarker of DNA damage to use jointly to other more specific tests, in biomonitoring workers exposed to mixture of bladder potential carcinogens such as rubber workers.

It could be important to monitor those patients who had positive results for at least two of the three tests (COMET, TRAP, and FISH), even if at the present the cystoscopy has not evidence of malignant pathologies, because the three tests may have identified very early steps of the disease, that is not yet detectable with the cystoscopic examination.

## 5. Conclusions 

Altogether, the findings show that the analysis of these biomolecular markers in urine cells could be potentially more accurate than conventional cytology in monitoring workers exposed to mixture of bladder potential carcinogens demonstrating the efficacy of a new generation noninvasive tests, to be used for early diagnosis of bladder cancer in subjects that were potentially in the past exposed to occupational carcinogenic agents in tires manufacturing.

## Figures and Tables

**Table 1:** Distribution of the studied population in different classes of risk on the basis of working information.

Tasks/working stage	Exposure time (years)	Exposure period	Class of risk	Number of subjects
Cabine electrician	<20	Not relevant	**1**	6
Machines maintenance-greasing	<10	Not relevant		
Tubes checking-inflating	<10	Not relevant		

Cabine electrician	>20		**2**	20
Machines maintenance-greasing	10–15			
Worker stages 1/2/4 (higher exposure risk)	<3	1962–1980		
Worker stages 1/2/4 (higher exposure risk)	<5	1981–2006		
Tubes checking-inflating	>10			

Machines maintenance-greasing	>15		**3**	29
Worker stages 1/2/4 (higher exposure risk)	3–5	1962–1980		
Worker stages 1/2/4 (higher exposure risk)	5–10	1981–2006		

Worker stages 1/2/4 (higher exposure risk)	5–10	1962–1980	**4**	30
Worker stages 1/2/4 (higher exposure risk)	>10	1981–2006		

Worker stages 1/2/4 (higher exposure risk)	>10	1962–1980	**5**	74

**Table 2:** Distribution of studied subjects for age, smoking habits, alcohol intake, drug assumptions, dietary, and statistical analysis of differences.

Variable	Modality	Ex-exposed group (*n* = 159)	Control group (*n* = 97)	Statistical significance of differences among groups *P* value
Age (years)		60 ± 12 Mean ± SD	57 ± 12 Mean ± SD	Mann Whitney test *P* = 0.003

Smoking	Nonsmokers	79	52	Chi square test *P* = 0.441
(8 missing exposed)	Ex-smokers	37	17	
2 missing controls	Smokers	35	26	

Recent drugs intake	No	127	88	Chi square test *P* = 0.1
(1 missing exposed)	Yes	31	9	

Diagnostic radiation exposure in the last six months	No	120	65	Chi square test *P* = 0.338
	Yes	39	32	

Alcohol assumption	No	5	10	Chi square test *P* < 0.001
(15 missing exposed)	Sometime	24	46	
6 missing controls	Yes	115	35	

Dietary habit (fruits and vegetables intake)	Sometime	77	34	Chi square test *P* = 0.014
(1 missing exposed)	Often	53	49	
3 missing controls	Usually	28	11	

Dietary habit (grilled and smoked food intake)	Sometime	24	43	Chi square test *P* < 0.001
(3 missing exposed)	Often	78	39	
3 missing controls	Usually	54	12	

**Table 3:** Comet test, TRAP, and FISH results on exfoliated urothelial cells of ex-workers distributed for class of risk and total ex-exposed group and controls.

Class of risk ex-exposed workers	*N*umber of subjects	Tail moment Mean ± SD	Number of subjects with DNA damage (%)	TRAP Median + SD	Number of subjects with TRAP positive (%)	Number of subjects with FISH and TRAP positive (%)
1	6	13.30 ± 10.72		33.50 ± 9.01		
2	20	14.32 ± 7.18		33.00 ± 13.21	1/26 (3.8%)	
3	29	11.58 ± 8.11	1/16 (6.2%)	35.83 ± 15.49	4/26 (15.4%)	1/14 (7.1%)
4	30	14.81 ± 10.97	2/16 (12.5%)	35.41 ± 13.08	3/26 (11.5%)	2/14 (14.3%)
5	74	20.39 ± 15.96	13/16 (81.3%)	41.00 ± 21.83	18/26 (69.3%)	11/14 (78.6%)

*P* value Kruskal -Wallis	*159 *	*0.023 *		*0.743 *		

Post hoc Bonferroni		5 > 3 **(0.022)**				

*P* value *χ* ^2^	*159 *		*0.054*		*0.111*	
Exact Fisher test *P* value			**0.020**		**0.065**	

Total ex-exposed ones	159	16.81 ± 13.23	16/159 (10%)	37 ± 18	26/159 (16%)	14/19 TRAP+ (74%)
Total controls	97	14.11 ± 7.18	2/97 (2%)	34 ± 20	15/97 (15%)	

*P* value Mann Whitney		**0.584**		**0.917**		

*P* value *χ* ^2^			**0.015**		**0.850**	

**Table 4:** Comparison of cystoscopy-biopsy with cytology, TRAP, FISH, and comet test results on exfoliated urothelial cells of ex-exposed workers distributed for class of risk.

Subject code	Class of risk	Cystoscopy-biopsy	Urinary			
			Cytology	TRAP	FISH	Comet
35	5	Dysplastic condition	Negative	Positive	Positive	Positive
44	5	Negative	Negative	Positive	Positive	Positive
65	5	Papillary UBC pTaG2	Negative	Negative	Not performed	Positive
86	5	Dysplastic condition	Negative	Positive	Positive	Positive
91	5	Negative	Negative	Positive	Positive	Negative
110	4	Negative	Negative	Positive	Positive	Positive
117	4	Negative	Negative	Positive	Not performed	Negative
148	5	Papillary infiltrating UBC pT3bN1G3	Hyper-dysplasia	Negative	Not performed	Positive
152	5	Phlogosis	Negative	Positive	Negative	Negative
178	5	Negative	Negative	Positive	Positive	Positive
207	5	Dysplastic condition	Negative	Positive	Positive	Positive
